# Management of renal extraskeletal mesenchymal chondrosarcoma

**DOI:** 10.1186/1471-2482-14-107

**Published:** 2014-12-15

**Authors:** Vitalie Gherman, Ciprian Tomuleasa, Catalina Bungardean, Nicolae Crisan, Victor-Dan Ona, Bogdan Feciche, Alexandru Irimie, Ioan Coman

**Affiliations:** Department of Urological and Robotic Surgery, Clinical Municipal Hospital in Cluj Napoca, Cluj Napoca, Romania; Research Center for Functional Genomics and Translational Medicine at the Iuliu Hatieganu, University of Medicine and Pharmacy, Cluj Napoca, Romania; Department of Hematology, Ion Chiricuta Cancer Center, Cluj Napoca, Romania; Department of Pathology, Clinical Municipal Hospital in Cluj Napoca, Cluj Napoca, Romania; Department of Urological Surgery, County Emergency Hospital in Satu-Mare, Cluj Napoca, Romania; Department of Surgery, Iuliu Hatieganu University of Medicine and Pharmacy, Cluj Napoca, Romania; Department of Surgery, Ion Chiricuta Comprehensive Cancer Center, Cluj Napoca, Romania; Department of Urology, Iuliu Hatieganu University of Medicine and Pharmacy, Cluj Napoca, Romania

**Keywords:** Extraosseous mesenchymal chondrosarcoma, Renal surgical management, Case report

## Abstract

**Background:**

Primary mesenchymal chondrosarcoma of the kidney is an extremely rare malignant tumor. To our best knowledge, only 9 such cases have been reported so far.

**Case presentation:**

In the current paper, we present the case of a 67 year-old patient with recurrent left lumbar pain, increased fatigability and intermittent macroscopic hematuria. He underwent a surgical resection of the left kidney and left hemicolon.

The pathological diagnosis was primary extraskeletal renal mesenchymal chondrosarcoma. Overall survival was 9 months, with pulmonary metastasis and local recurrence at 6 months. The management of the patient is described, from the initial differential diagnosis, after the first clinical examination to the surgical resection, with a special emphasis on the surgical procedures that were carried out.

**Conclusion:**

Extraskeletal chondrosarcoma of primary origin in the kidney are extremely rare tumors with a highly malignant potential and very poor prognosis. Because the role of chemotherapy or radiation therapy has not been evaluated properly yet, we underline the importance of surgery in the management of such cases as the main and best approach to achieve clinical remission and long-term survival, provided the patient is referred to a surgical consult in time.

## Background

Mesenchymal chondrosarcomas (MC) were initially described by Lichtenstein and Bernstein more than 50 years ago as rare tumors that affect the bone tissue [1], other case reports followed from the surgical department of the Mayo Clinic in Rochester, USA [2]. Pathologists separate mesenchymal chondrosarcomas from other classical or de-differentiated chondrosarcomas due to a specific histological pattern formed of highly undifferentiated small round cells, admixed with islands of well-differentiated hyaline cartilage [3, 4]. Epidemiology data state that MCs represent about 2% of all chondrosarcoma cases and usually arise in the osseous tissue of the adult skeleton [5]. Nevertheless, several reports show that MCs may be diagnosed in other soft tissues that are not directly associated to the bone, such as the axillary region, the heart, the thyroid gland, the pancreas, orbital region or even the kidney [6–11]. Such diagnoses are challenging due to their rarity and paucity of reports.

In the current paper, we report description of the surgical management of a renal MC, an extremely rare condition to our knowledge, with only nine other cases having been previously reported [12–17].

## Case presentation

### Methods

#### Clinical presentation

**Figure 1:**
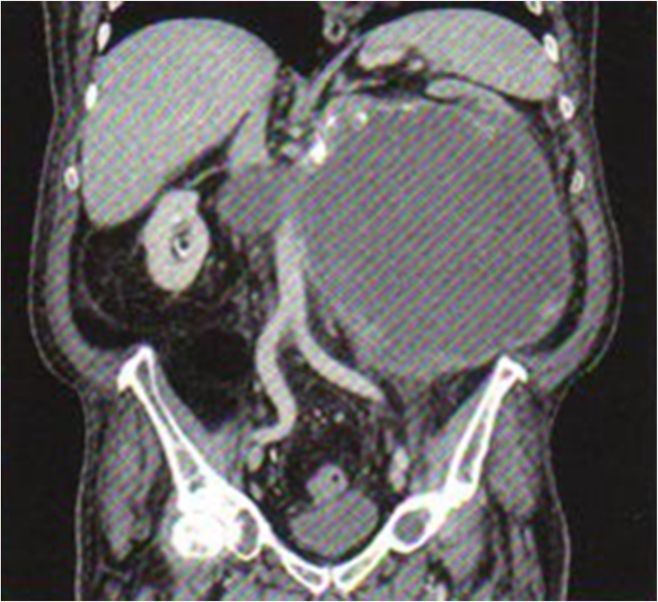
**Abdominal CT sowing the huge renal tumor.**

In the current paper, we present the case of a 67 year-old patient who was hospitalized with recurrent left lumbar pain, increased fatigability and intermittent macroscopic hematuria. On clinical examination, a large tumor was noticed on the left side. The routine laboratory tests showed that out patient had anemia and hypercalcemia. Ultrasonography revealed a giant, heterogeneous tumor mass of the left retroperitoneum, with a poor vascular signal, as well as multiple hyperechogenous regions. The abdominal computer tomography (CT) (Figure 1) confirmed our previous clinical diagnosis, showing a huge left renal tumor of 30 cm in its largest diameter. The excretory function of the left kidney was inexistent, with no evidence of retroperitoneal lymphadenopathy, as well as no evidence of a thrombus of the renal vein or secondary metastatic dissemination. Routine Rx of the chest did not reveal the presence of lung metastasis.

**Figure 2:**
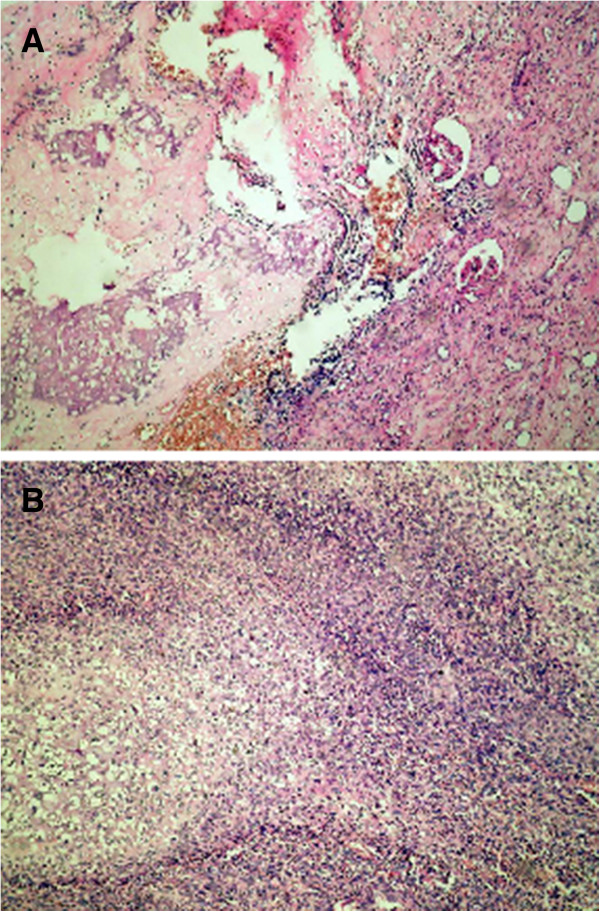
**H&E staining.** Biphasic tumor **(A)**. Hemangioperycitomatous pattern **(B)**.

As safety and accuracy of percutaneous biopsy was recently reported by Volpe et al [18], we have performed a fine needle aspiration biopsy of this radiological undetermined renal mass, in order to establish the diagnosis and to assess the potential role of the neoadjuvant chemotherapy. The pathological examination showed well differentiated, benign appearing cartilaginous tissue, raising the suspicion of a chondrosarcoma, osteosarcoma or another cartilage producing tumor (Figure 2).

Within the tumour board of the Cancer Center, the oncologist sustained the idea of neoadjuvant chemotherapy with cisplatin and doxorubicin. Still, the performance status of the patient did not allowed optimal clinical management, as tumour progression was assessed, and surgery was imperative. Soon after the percutaneous biopsy was done, the general condition of the patient began to worsen, as weight loss was noticed along with a diagnosis of sub-occlusive syndrome. The surgical indication was of a left radical nephrectomy and left hemicolectomy, as further described.

#### Surgical management

The first step was to place the patient in a supine position in an extended bed at about 30 degrees below the lumbar space. The operation begun with a bilateral subcostal incision (Chevron incision) completed with a left para-rectal extension and an intercostal branch. The inspection of the abdominal cavity revealed a giant left renal mass of 35 cm in the largest diameter, with a cystic appearance and liquid content, which was highly adherent to the mesocolon. Palpation and detailed inspection of the other intraperitoneal organs did not reveal any noticeable, macroscopic metastases, except the mesocolon, surrounding lymph nodes were not enlarged.

Because of its volume, and severely modified loco-regional anatomy, manipulation was difficult and the excision of the tumour in a “non-touch technique”, with the primary ligation of the vascular supply, was virtually impossible. Excision was only possible after the evacuation of the intratumoral content of about 2500 cc of serosanguinous, viscous and gelatinous liquid, using all the available methods to minimize intraperitoneal tumoral spillage (high-power vacuum, hypertonic solutions, protection of the intra-abdominal organs with sterile, watertight materials).

**Figure 3:**
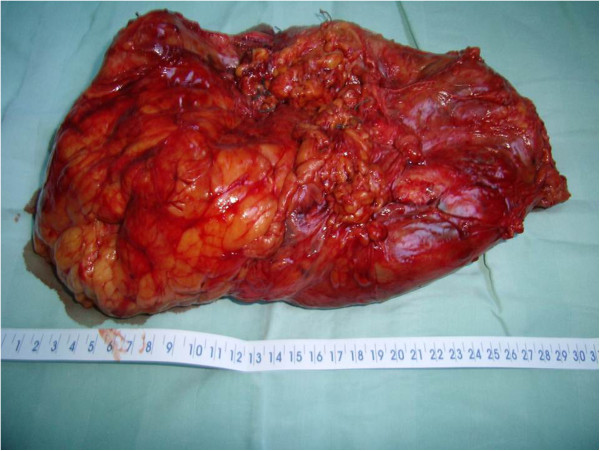
**The giant tumoral kidney of 35 cm.**

As the colon was very adherent to the medial side of the tumor, the dissection of the mesocolon from the Gerota’s fascia, as initially planned, was virtually impossible. The intraoperative decision was to perform a left hemicolectomy with a termino-terminal anastomosis of the colon. After this step was finished successfully, having the resected colon reflected laterally, the surgeons were able to correctly identify the elements of the renal hilum. First, the renal vein was mobilized completely by ligating and dividing all its tributaries. The next procedure was to identify the renal artery, followed by its ligation and dividing. Similar management was performed for the renal vein and for the ureter. The kidney was mobilized outside the Gerota’s fascia, using both sharp and blunt dissections, together with the ipsilateral adrenal gland and approximately 20 cm of the mesocolon (Figure 3).

### Results

**Figure 4:**
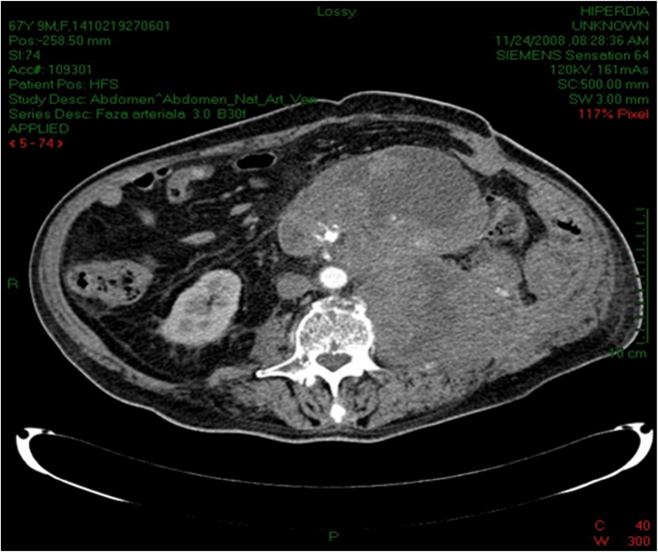
**CT scan 6 months after surgery.** Massive local relapse.

The total time of the surgery was 210 minutes, and the patient had a blood loss of approximately 2000 ml. In postoperative patient received broad spectrum antibiotics and 3 units of blood. After the surgical intervention was finished successfully, the recovery of the patient was slow, but favorable. He was discharged 10 days after surgery. As the patient had a high risk for recurrence due to the positive surgical margins and tumour aggressiveness, the clinical management consisted of an intensified follow-up algorithm, in accordance with the guidelines and radiation regulations [19, 20]. The first CT performed at two months after the surgery, showed no recurrence or metastasis. CT scan performed at six months after the surgery showed pulmonary metastasis and a massive local relapse (extending from the psoas muscle, encompassing and displacing aorta (Figure 4).

After the consult of the medical oncologist, it was confirmed that the patient had a stage IV tumor and no salvage chemotherapy was appropriate, taking into considerations his comorbidities and poor performance status. Supportive care and pain management was recommended and pursued until the patient died three more months later, at approximately 9 months after the surgery.

#### Pathology diagnosis

The whole specimen was fixed in 10% formalin. After fixation, multiple specimens where paraffin embedded sectioned 4 micrometers thick and coloured with haematoxylin-eosin (H&E). Microscopical examination revealed a pushing biphasic tumor, composed of large sheets of undifferentiated round or slightly spindle-shaped cells arranged in a hemangioperycitomatous pattern, and islands of well differentiated cartilage, with no transition zone between them. The histological picture included foci of osteoid, ossification and calcification, haemorrhage and necrosis. The remaining renal parenchyma showed areas of coagulative necrosis; the tumour did not invade the renal pelvis, but it was extended beyond perinephric tissues into Gerota’s fascia, and paranehric fat, with no evidence of infiltration into renal vein (pT4NxM0V0R1). As tumour spread also involved the paranephric fat, surgeons were not able to obtain completely clean surgical margins (R0). The colon and the adrenal were free of tumour. The concluding diagnosis was extrascheletal MC of the kidney.

### Discussions

Extraskeletal chondrosarcomas are rare neoplasms, far less common in comparison with the osseous ones [21] and pathologists separate these cases into two different subtypes: myxoid and mesenchymal. In contrast with the myxoid subtypes, the mesenchymal chondrosarcomas are diagnosed even in fewer cases and are very aggressive [22], with respective 5- and 10-year survival rates of 54.6% and 23.7% [6]. As previously stated, literature search limited to the English, French and German language unearthed very few cases of MC, especially for the ones located in the kidney. These cases are different from a typical chondrosarcoma in several aspects. First, they are of extraskeletal origin, so a thorough skeletal search is essential for a correct differential diagnosis between a metastatic MC and one with a primary extraskeletal localization. MC show a predilection for middle aged males, whereas extraskeletal MC are diagnosed more frequently in females and have a higher incidence in the nervous system for the ones in their 20s and other soft tissues above the age of 40 [23].

Because of their rarity and lack of appropriate studies and clinical trials to assess the best treatment of choice, there are no international accepted guidelines for the management of primary renal MC. Huvos et al. further divided the patients into two groups based on the pathological diagnosis: undifferentiated small cell types and hemangiopericytomatoid type [24], but his protocols are valid for primary skeletal tumors and no guidelines were mentioned for the cases of extraskeletal origin. Later, Knott et al. and Rushing et al. suggest some guidelines regarding the wide margins for the surgical resection for these very rare cases [25], but he did not mention any standard-of-care for primary renal MC.

Systemic chemotherapy or radiation therapies have an unknown role for these cases due to absence of objective evidence [26]. Still, since the excision was not performed following the steps of the “non-touch technique” [27], and because tumoral spillage represented a real possibility, the role of intraperitoneal chemotherapy [28] should have been assessed, as this method showed encouraging results in other types of cancer. Data are scarce for extraskeletal MC and even less data is available to evaluate the best treatment option for the ones that have a renal primary localization. Our study is very rare and the interdisciplinary consensus panel of the Clinical Municipal Hospital and Ion Chiricuta Comprehensive Cancer Center suggested that the best management is neoadjuvant chemotherapy followed by a wide surgical resection, reported in the current paper. This makes our case one of the very few reports that try to establish a standard-of-care for future patients diagnosed with similar tumors.

Mesenchymal chondrosarcomas are highly malignant and have a propensity to metastasize especially to the lungs [5], as was the case in our patient. This makes the accurate diagnosis the first important step, based on the characteristic clinical, radiological and histopathological features. Furthermore, based on our experience, we can emphasize the importance of surgery in the management of such cases as the main and best approach to achieve clinical remission and long-term survival, provided the patient is referred to a surgical consult in time.

## Conclusion

Extraskeletal chondrosarcoma of primary origin in the kidney are extremely rare tumors with a highly malignant potential and very poor prognosis. Because the role of chemotherapy or radiation therapy has not been evaluated properly yet, the main treatment-of-choice is a wide surgical resection with clean margins, after an early and correct diagnosis has been made.

## Consent

Written informed consent was obtained from the patient for publication of this Case report and any accompanying images. A copy of the written consent is available for review by the Editor of this journal.
